# Jelly Fig (*Ficus awkeotsang* Makino) Exhibits Antioxidative and Anti-Inflammatory Activities by Regulating Reactive Oxygen Species Production via NFκB Signaling Pathway

**DOI:** 10.3390/antiox11050981

**Published:** 2022-05-17

**Authors:** Meng-Jin Lin, Ping Lin, Kuo-Ching Wen, Hsiu-Mei Chiang, Mei-Chun Lu

**Affiliations:** 1 Miaoli District Agricultural Research and Extension Station, Council of Agriculture, Executive Yuan, Miaoli 363-201, Taiwan; lmj@mdais.gov.tw; 2 Department of Cosmeceutics, China Medical University, Taichung 406-040, Taiwan; u9815010@cmu.edu.tw (P.L.); kcwen0520@mail.cmu.edu.tw (K.-C.W.); hmchiang@mail.cmu.edu.tw (H.-M.C.)

**Keywords:** antioxidant, anti-inflammatory, plant extract, reactive oxygen species (ROS), nuclear factor-κB (NFκB), inducible nitric oxide synthase (iNOS), cyclooxygenase-2 (COX-2), interleukin-6 (IL-6)

## Abstract

Antioxidant and anti-inflammatory activities of *Ficus awkeotsang* Makino extract (FAE) on Hs68 fibroblasts and BALB/c nude-mouse models are evaluated in this study. FAE was found to be non-toxic and showed high levels of DPPH, H_2_O_2_, and hydroxyl radical scavenging abilities; a ferrous chelating capacity; as well as ferric-reducing antioxidant capability. The antioxidant activity of FAE was strongly associated with polyphenolic content (flavonoids at 10.3 mg QE g^−1^ and total phenol at 107.6 mg GAE g^−1^). The anti-inflammatory activity of FAE and the underlying molecular mechanisms were also investigated. The a* value of the mouse dorsal skin after treatment with FAE at 1.5 mg/mL in addition to chronic UVB exposure was found to decrease by 19.2% during a ten-week period. The anti-inflammatory effect of FAE was evidenced by the decreased accumulation of inflammatory cells and skin thickness. Expression levels of UVB-induced inflammatory proteins, including ROS, NF-κB, iNOS, COX-2, and IL-6, were significantly reduced upon FAE treatment in vitro and in vivo. Collectively, our results suggest that the inhibition of ROS and UVB-induced activation of the NF-κB downstream signaling pathway by FAE, indicating considerable potential as a versatile adjuvant against free radical damage in pharmaceutical applications.

## 1. Introduction

The skin is the largest organ of the human body and the first line of defense against pathogens and environmental attacks [1]. Solar ultraviolet B (UVB) irradiation (wavelengths at 290–320 nm) is a common environmental cause of cutaneous tissue injury [2,3]. UVB irradiation also induces DNA damage, oxidative stress, and inflammatory processes in keratinocytes, and thereby results in symptoms, such as skin inflammation, photo-aging, and photo-carcinogenesis [4]. UVB-induced damage is largely attributed to the presence of reactive oxygen species (ROS) that are generated via the transfer of electromagnetic energy from UVB radiation to the mitochondrial electron transport chain during cellular respiration [5]. ROS are considered to be regulators of cellular signaling pathways involved in aerobic metabolism, yet excessive ROS levels can cause diseases, such as cancer, neurological disorders, cardiovascular diseases, rheumatoid arthritis, diabetes mellitus, cataracts, and various pulmonary diseases [6,7].

Plant extracts often include ROS scavenger compounds that are effective in various ailments [8]. Herbal formulas have lately seen a resurgence in cosmetology [9]. Despite the widespread use of herbal medicine as complementary and alternative therapies for skin conditions and cancer, our knowledge of the risks associated with the long-term use of non-edible, wild, or stranger plants is limited. For example, rhododendrol, 4-(4-hydroxyphenyl)-2-butanol, a compound extracted from *Acer nikoense* and *Betula platyphylla*, was withdrawn from the market in 2013 due to cases of chemical leukoderma as a result of cytotoxicity induced by oxidative metabolites [10,11]. The development of efficacious and safe plant extracts thus remains an important objective for cosmetic and clinical applications.

Jelly fig (*Ficus awkeotsang* Makino) is an indigenous vine species and a member of the Moraceae family. Jelly fig grows in the mountainous areas of Taiwan at 800 to 1800 m above the sea level. Numerous small achenes are obtained from the fruit of jelly fig after pollination and harvest, and pectin is extracted from dried seeds to produce jelly by mixing with water, which has become a popular traditional pectin-rich dessert for over 150 years in Asia [12,13]. The extract from jelly fig seeds is known to have apoptotic and anti-proliferative effects on leukemic and colorectal cancer cells [14,15,16]. It is also known for its ability to inhibit surface antigen expression in human hepatitis B virus [17]. Moreover, *Ficus* species, including fig (*Ficus carica* L.), Chinese banyan (*Ficus microcarpa* L.), and cluster fig (*Ficus racemose* L.), are excellent sources of antioxidants, whose free radical scavenging effects have been demonstrated previously [18,19,20,21,22,23]. Nonetheless, it is uncertain if *Ficus awkeotsang* Makino extract (FAE) has an antioxidant activity. Hence, the aim of this study is to determine the antioxidant and anti-inflammatory effects of FAE, and the associated molecular mechanisms.

## 2. Materials and Methods

### 2.1. Materials

*Ficus awkeotsang* Makino was cultured in our institution (Taiwan Jelly Fig Germplasm Bank”, Miaoli District Agricultural Research and Extension Station, Miaoli, Taiwan) for achene production. The extract of *Ficus awkeotsang* Makino (FAE) was modified according to procedures previously described [24]. Briefly, mature fruits were peeled and dried after harvest, and the dried achenes were scratched from the syconium wall. Then, 1 g of jelly fig achene was immersed in 100 mL of deionized water and sonicated for 1 h. The mixtures were stirred at room temperature for 24 h before filtering using filter paper (Whatman Grade No.1, Dassel, Germany). The extracts were stored at 4 °C until further analysis.

### 2.2. Cell Cytotoxic Activity Assay

Human skin fibroblasts (Hs68 cells) (Bio-resource Collection and Research Centre, Hsinchu, Taiwan) were cultured in Dulbecco’s modified Eagle’s medium (DMEM) supplemented with 10% fetal bovine serum and 100 U/mL penicillin/streptomycin at 5% CO_2_ and 37 °C. Cell viability was measured using 3-(4, 5-dimethylthiazol-2-yl)-2,5-diphenyltetrazolium bromide (MTT) (USB Corporation, Cleveland, OH, USA) after treatment with 0.25–2.0 mg/mL FAE, and also examined using a microplate reader at an excitation wavelength of 570 nm (Sunrise, Tecan, Salzburg, Austria) according to the manufacturer’s instructions.

### 2.3. In Vitro Antioxidant Measurement

#### 2.3.1. Ferrous Ion Chelating Activity

The chelation of ferrous ions was determined using the ferrozine assay with slight modifications, as previously described [6,25]. A series of FAE concentrations was added to a 2 mM FeCl_2_ solution. Then, the 5 mM ferrozine was added to the solution, and the mixture was shaken vigorously. The absorbance of the mixture was determined at 562 nm using an ELISA reader (Synergy HTX, BioTek Instruments, Winooski, VT, USA). EDTA was used as a positive control. The results were expressed as the percentage inhibition of the formation of the ferrozine-Fe^2+^ complex. The ferrous chelation activity was calculated using Equation (1) as follows:

(1) $$\mathrm{Ferrous}\;\mathrm{ion}\;\mathrm{chelating}\;\mathrm{activity}\left(\%\right)=(\frac{A_{\mathrm{control}\;\mathrm{at}\;562\;\mathrm{nm}}-A_{\mathrm{sample}\;\mathrm{at}\;562\;\mathrm{nm}}}{A_{\mathrm{control}\;\mathrm{at}\;562\;\mathrm{nm}}})\times 100$$

#### 2.3.2. Ferric-Reducing Antioxidant Capability

The ferric-reducing antioxidant capability of FAE was measured using the potassium ferricyanide-ferric chloride method [26]. Briefly, 0.2 mL of FAE at different concentrations, 2.5 mL of phosphate buffer (0.2 M, pH 6.6), and 2.5 mL of potassium ferricyanide K_3_Fe(CN)_6_ (1%) were mixed and incubated at 50 °C for 20 min. The reaction was stopped by adding 2.5 mL of 10% (*w*/*v*) trichloroacetic acid, and then centrifuged at 1000 g for 10 min. Finally, 2.5 mL of the supernatant was mixed with 2.5 mL of deionized water and 0.5 mL ferric chloride (0.1%). The absorbance of the reaction solution was determined at 700 nm using a microplate reader (Sunrise, Tecan, Salzburg, Austria). Ascorbic acid and distilled water were used as the positive and negative controls, respectively. The reducing capability was calculated using Equation (2) as follows:

(2) $$\mathrm{Ferric}\;\mathrm{reducing}\;\mathrm{antioxidant}\;\mathrm{capability}\left(\%\right)=\left(\frac{A_{\mathrm{control}\;\mathrm{at}\;700\;\mathrm{nm}}-A_{\mathrm{sample}\;\mathrm{at}\;700\;\mathrm{nm}}}{A_{\mathrm{control}\;\mathrm{at}\;700\;\mathrm{nm}}}\right)\times 100\%$$

#### 2.3.3. Hydrogen Peroxide Scavenging Activity

Hydrogen peroxide scavenging activity of FAE was measured spectrophotometrically, as previously described [27]. FAE dissolved in methanol at various concentrations was added to a solution, including 20 mM H_2_O_2_ in PBS. The resulting mixture was then incubated for 10 min in the dark at room temperature. Absorbance was determined at 230 nm using a microplate reader (Synergy HTX, BioTek Instruments, Winooski, VT, USA). The hydrogen peroxide scavenging activity was calculated using Equation (3) as follows:

(3) $$\mathrm{Hydrogen}\;\mathrm{peroxide}\;\mathrm{scavenging}\;\mathrm{activity}\;\left(\%\right)=\left(\frac{A_{\mathrm{control}\;\mathrm{at}\;230\;\mathrm{nm}}-A_{\mathrm{sample}\;\mathrm{at}\;230\;\mathrm{nm}}}{A_{\mathrm{control}\;\mathrm{at}\;230\;\mathrm{nm}}}\right)\times 100$$

#### 2.3.4. DPPH Radicals Scavenging Activity

The α, α-diphenyl-β-picrylhydrazyl (DPPH) radical scavenging activity of FAE was measured according to a previously described method [28]. Briefly, serial dilutions of FAE aliquots were mixed with 900 μL of methanol and 5.0 μL of a methanolic DPPH solution (10 mmol L^−1^). The reaction was allowed to run for 30 min at room temperature in the dark, and the absorbance was measured using a microplate reader at 517 nm (Sunrise, Tecan, Salzburg, Austria). Ascorbic acid was used as the positive control. The DPPH radical scavenging activity was calculated according to Equation (4) as follows:

(4) $$\mathrm{DPPH}\;\mathrm{radical}\;\mathrm{scavenging}\;\mathrm{activity}\;\left(\%\right)=\left(\frac{A_{\mathrm{control}\;\mathrm{at}\;517\;\mathrm{nm}}-A_{\mathrm{sample}\;\mathrm{at}\;517\;\mathrm{nm}}}{A_{\mathrm{control}\;\mathrm{at}\;517\;\mathrm{nm}}}\right)\times 100$$

#### 2.3.5. Hydroxyl Radical Scavenging Activity

Hydroxyl radical scavenging activity of FAE was determined using a previously reported method [29]. First, a solution was prepared by mixing FAE, deoxyribose, EDTA, FeCl_3_, KH_2_PO_4_-KOH buffer, H_2_O_2_, and ascorbic acid. Then, TCA and TBA were added, and the resulting mixture was incubated for 15 min at 100 °C. The absorbance of the mixture was determined at 532 nm using a microplate reader (Synergy HTX, BioTek Instruments, Winooski, VT, USA). The hydroxyl radical scavenging activity of FAE was reported as the percentage inhibition of deoxyribose degradation. The hydroxyl radical scavenging activity was calculated according to Equation (5) as follows:

(5) $$\mathrm{Hydroxyl}\;\mathrm{radical}\;\mathrm{scavenging}\;\mathrm{activity}\left(\%\right)=\left(\frac{A_{\mathrm{control}\;\mathrm{at}\;532\;\mathrm{nm}}-A_{\mathrm{sample}\;\mathrm{at}\;532\;\mathrm{nm}}}{A_{\mathrm{control}\;\mathrm{at}\;532\;\mathrm{nm}}}\right)\times 100$$

### 2.4. Measurement of In Vitro Inflammatory Activity

Cells covered with PBS were exposed to 40 mJ/cm^2^ UVB (emission peak at 302 nm) using a CL-1000 M UV crosslinker (UVP, Upland, CA, USA), and the intracellular oxidative stress of Hs68 cells was quantified using DCFDA fluorogenic dye (Sigma-Aldrich, Saint Louis, MO, USA). Fluorescence was detected using a microplate reader (Synergy HTX, BioTek Instruments, Winooski, VT, USA) at emission and excitation wavelengths of 520 and 488 nm, respectively. For immunofluorescence staining, HS68 cells were fixed on coverslips with 4% paraformaldehyde after exposure to 40 mJ/cm^2^ UVB. The slips were blocked with 5% skim milk in Tris-buffered saline (pH 7.6) containing 0.3% Triton X-100 and incubated with the following primary antibodies: anti-NFκB antibody (Cell Signaling, Danvers, MA, USA) and anti-4, 6-diamidino-2-phenylindole (DAPI) antibody (Cell Signaling, Danvers, MA, USA) for 30 min at 20 ± 1 °C. Following washing with PBS, the slips were incubated with the secondary-antibody anti-rabbit immunoglobulin (IgG) (Alexa Fluor 488, Invitrogen, Carlsbad, CA, USA) for 30 min at 20 ± 1 °C. The cover slips were counterstained with ProLong Gold anti-fade reagent and DAPI (Thermo Fisher Scientific, Waltham, MA, USA), and images were captured using a fluorescence microscope (Leica DMIL, Wetzlar, Germany).

### 2.5. Immunoblotting Assay

RIPA lysis buffer (Thermo Fisher Scientific Inc., Waltham, MA, USA) was used to extract total protein from Hs68 cells. The extraction was performed on a gradient sodium dodecyl sulfate (SDS)–sulfate-polyacrylamide gel, and transferred onto a polyvinylidene difluoride membrane. Membranes were blocked with 5% skim milk in Tris-buffered saline (pH 7.6) containing 0.3% Triton X-100, and incubated with the following primary antibodies: anti-cyclooxygenase-2 (COX-2) antibody (Genetex, Irvine, CA, USA), anti-inducible nitric oxide synthase (iNOS) antibody (Genetex, Irvine, CA, USA), and anti-actin antibody (Genetex, Irvine, CA, USA) at 20 ± 1 °C. Following washing with TBST, membranes were incubated with a horseradish peroxidase (HRP)-conjugated secondary antibody (Alexa Fluor 488; Invitrogen, Carlsbad, CA, USA) for 2 h at 20 ± 1 °C, followed by membrane incubation in an enhanced chemiluminescent (ECL) detection system (LAS-4000, Fujifilm, Tokyo, Japan) for target-antigen visualization. The intensity of the targeted protein bands was quantified and normalized to the actin expression area using the ImageJ 1.8.0v software (National Institutes of Health, Bethesda, MD, USA).

### 2.6. Determination of In Vivo Inflammatory Activity

The murine inflammatory assay was approved by the Institutional Animal Care and Use Committee of China Medical University (IACUC Approval No: CMUIACUC-2016-421). Five-week-old wild-type female BALB/c nude mice were purchased from the National Laboratory Animal Center (Taipei, Taiwan), and acclimatized to the animal facility for one week. Mice were provided free access to water and food in polycarbonate shoebox cages with hardwood bedding at 21 ± 1 °C in a 12 h/12 h light/dark cycle. Mice were exposed to 180 mJ/cm^2^ UVB irradiation 3 times a week for 1 min per exposure, and then treated with 100μL vehicle-polyethylene glycol (PEG) or 100 μL FAE on the exposure area per time for 10 weeks. Mice were divided into the following treatment groups: A, Normal group; B, UVB group; C, UVB + vehicle; D, UVB + 1.5 mg/mL FA extract; and E, UVB + 2.5 mg/mL FAE. The a* value was detected using a spectrophotometer (SCM-108, Laiko company, Tokyo, Japan) according to the formula provided by manufacturer’s instructions for 2, 4, 6, 8, and 10 weeks.

### 2.7. Immunofluorescence Staining

Human skin fibroblasts (Hs68 cells) were grown on a cover slip and treated with 0.25–2.0 mg/mL FAE after UVB irradiation. After 24 h, the cells were fixed with 4% paraformaldehyde and washed with PBS. They were then blocked with MPBS (containing 5% non-fat milk solution and 0.3% Triton X-100) for 30 min. PBS was again used to wash the coverslip, and incubation was performed with a primary antibody for 30 min and then with a secondary antibody, anti-rabbit immunoglobulin (IgG) (Alexa Fluor 488, Invitrogen, Carlsbad, CA, USA). Coverslips were then washed with PBS to remove unbound secondary antibodies, and counterstained using ProLong Gold anti-fade reagent with 4′,6-diamidino-2-phenylindole. A fluorescence signal was observed using a confocal laser scanning microscope (Leica Microsystems, Wetzlar, Germany).

### 2.8. Total Phenol and Flavonoids Assay

Total phenol content was determined using the modified Folin–Ciocalteu method [30]. Briefly, 1 mL gradient dilutions of FAE (0.25–2.0 mg mL^−1^) were mixed with 2.5 mL of 10% (*w*/*v*) Folin–Ciocalteu reagent and 2 mL of 75% Na_2_CO_3_ for 30 min at room temperature, followed by an absorption measurement at 765 nm using a microplate reader (Sunrise, Tecan, Salzburg, Austria). Gallic acid was used as the standard to create a standard curve (0–100 μg mL^−1^), and the total phenol content was examined as mg of gallic acid equivalents of dry plant extract (mg GAE g^−1^ DW). Flavonoid content was measured as previously described [31]. Briefly, 0.2 mL of 10% (*w*/*v*) AlCl_3_-methanol solution, 0.2 mL of 1 M potassium acetate, and 5.6 mL distilled water were added to 1 mL of FAE solution (0.25–2.0 mg mL^−1^). The resulting mixture was incubated for 30 min at room temperature, and the absorbance was measured at 405 nm. Quercetin was used as the standard to prepare a standard curve (0–100 μg mL^−1^) to obtain mg quercetin acid equivalents of dry plant extract (mg QE g^−1^DW).

### 2.9. Nuclear Magnetic Resonance Spectroscopy (NMR) and High-Performance Liquid Chromatography (HPLC) Analysis

Nuclear magnetic resonance (NMR) spectral analysis was performed to determine the structure of the jelly fig extract using a Varian Unity Inova 500 MHz spectrometer (Varian, Palo Alto, CA, USA) at 6250 Hz for ^1^H. Chemical shifts (δ) were expressed in ppm. Tetramethylsilane (TMS, δ 0 ppm) was used as an internal standard [32]. A high-performance liquid chromatography (HPLC) (Shimadzu SCL-10A VP) coupled with a SPD-M 10A VP diode array detector was performed for the qualitative determination of compounds in the FAE. Samples were separated by a reverse-phase column (Phenomenex C18, 5 μm, 250 × 100 mm) maintained at 25 °C. The flow rate was 1 mL/min. The detection wavelength was set at 250 nm.

### 2.10. Statistical Analysis

Each experiment was repeated at least three times (*n* = 3). Statistical analyses were performed using the SAS-EG 7.1 software (SAS Enterprise Guide, SAS Institute Inc., Cary, NC, USA). One-way ANOVA and Tukey’s post hoc test were used to determine statistically significant differences (* *p* < 0.05, ** *p* < 0.01, and *** *p* < 0.001) between data obtained from the control and test samples. Error bars were used to denote standard deviations from the mean (SD).

## 3. Results

### 3.1. Cytotoxic Activity

**Figure 1:**
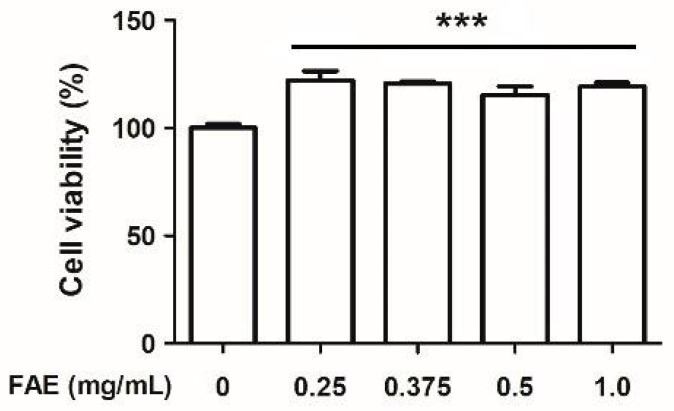
The cytotoxic activity of *Ficus awkeotsang* Makino extract (FAE) in Hs68 cells for 24 h. Significant differences between control (0 mg/mL FAE) and other treatments were determined using Turkey HSD test. *** *p* < 0.001; *n* = 3; all error bars represent the standard deviation of the mean.

Human skin fibroblast (Hs68 cell) is a standard cellular model of the human skin layer; it was used to determine if FAE could enhance the protective effect after UVB irradiation in a condition that closely resembled the physiological conditions. First, we determined the cytotoxicity of FAE in the Hs68 cell for further application. The viability of Hs68 cells following FAE application was evaluated using the standard MTT proliferation assay at 37 °C for 24 h (Figure 1). The lowest level of cell survival was observed with control (PBS) as 100.0%, followed by 0.25, 0.375, 0.5, and 1.0 mg/mL of FAE application, which yielded 122.2%, 120.6%, 115.4%, and 119.2%, respectively. No cytotoxicity was detected upon co-incubation with FAE, which significantly (*p* < 0.001) increased the viability of Hs68 cells, demonstrating the biocompatibility of FAE.

### 3.2. Antioxidant Capacity

**Figure 2:**
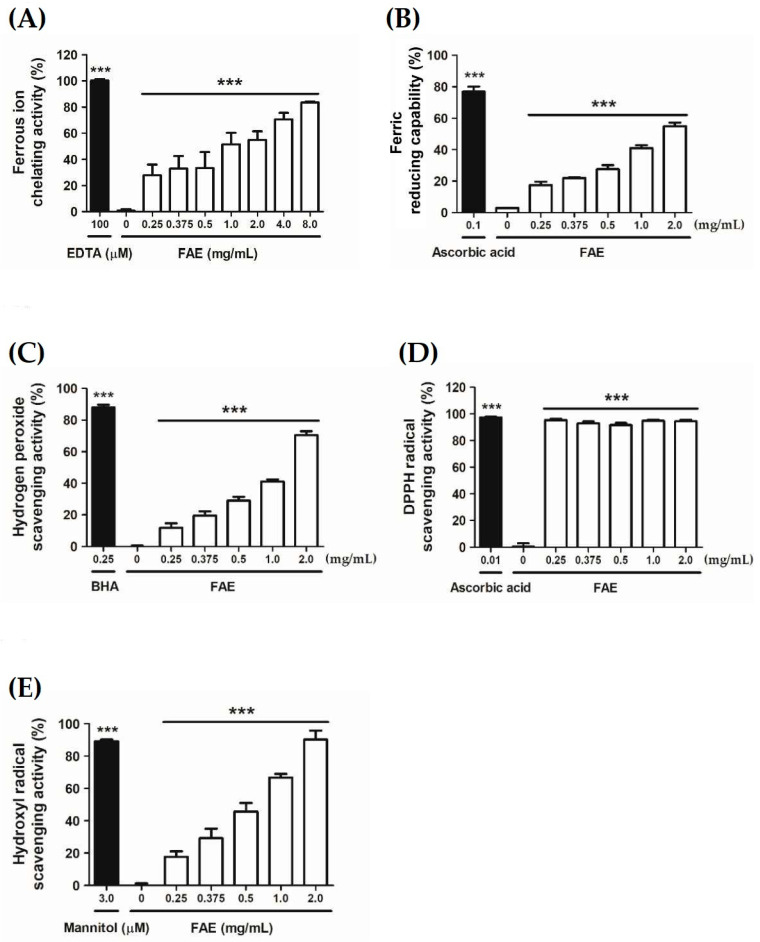
The antioxidant capacity of *Ficus awkeotsang* Makino extract (FAE). (**A**) Ferrous chelating activity, (**B**) ferric-reducing antioxidant capability, (**C**) DPPH radical scavenging activity, (**D**) H_2_O_2_ scavenging activity, and (**E**) hydroxyl radical scavenging activity. Statistical significance of the differences between negative control (0 mg/mL FAE treatments), positive standard (black bar), and other treatments were determined using Tukey’s HSD test. *** *p* < 0.001; *n* = 3; all error bars represent the standard deviation of the mean.

We investigated the cytotoxic and antioxidative effects of various FAE concentrations on Hs68 cells to determine the optimum FAE concentration for inflammatory activity. The findings related to the antioxidant capacity of FAE, namely, the ferrous chelating activity, ferric-reducing capability, and H_2_O_2_ scavenging activity were found to increase by 27.1–82.7% (*p* < 0.001, Figure 2A), 14.6–52.0% (*p* < 0.001, Figure 2B) and 11.8–70.5% (*p* < 0.001, Figure 2C), respectively, upon treatment with different FAE concentrations (0.25–8.0 mg/mL). Interestingly, the DPPH radical scavenging activity of ascorbic acid (10 μg/mL) was found to be 97.5%, which is similar to that of FAE (91.6–94.4%), irrespective of the applied concentration (Figure 2D). Further analysis showed that 2.0 mg/mL FAE yielded a higher hydroxyl radical scavenging activity than 3 μM mannitol (Figure 2E).

**Figure 3:**
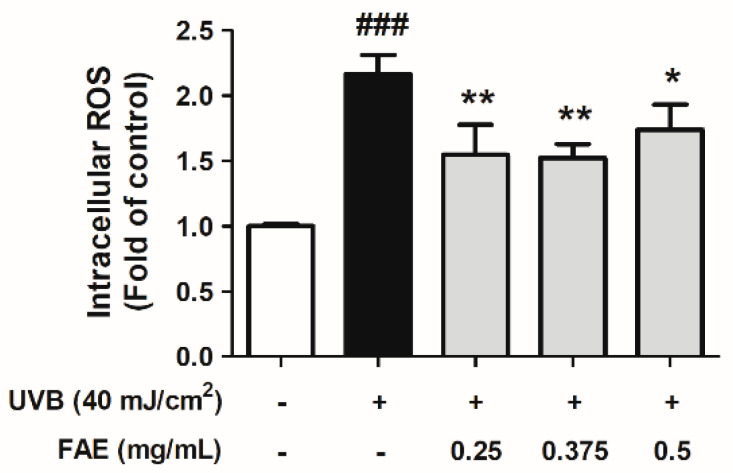
Inhibitory effects of *Ficus awkeotsang* Makino extract (FAE) on production of intracellular reactive oxygen species (ROS) in UVB-irradiated Hs68 cells. Significant difference with control (white bar): ### *p* < 0.001; significant difference with UVB-exposure group (black bar): * *p* < 0.01, ** *p* < 0.05. Tukey’s HSD test was used to determine statistical significance. *n* = 3; all error bars represent the standard deviation of the mean.

The fold change in intracellular reactive oxygen species (iROS) was evaluated after UV irradiation. A significant increase (2.17-fold, *p* < 0.001) in iROS level in UVB-irradiated Hs68 cells was observed compared to the control. However, the level of change in the iROS level was decreased to 1.55 fold, 1.53 fold, and 1.74 fold, upon FAE treatment at 0.25, 0.375, and 0.5 mg/mL, respectively (Figure 3).

### 3.3. In Vitro Anti-Inflammatory Activity

**Figure 4:**
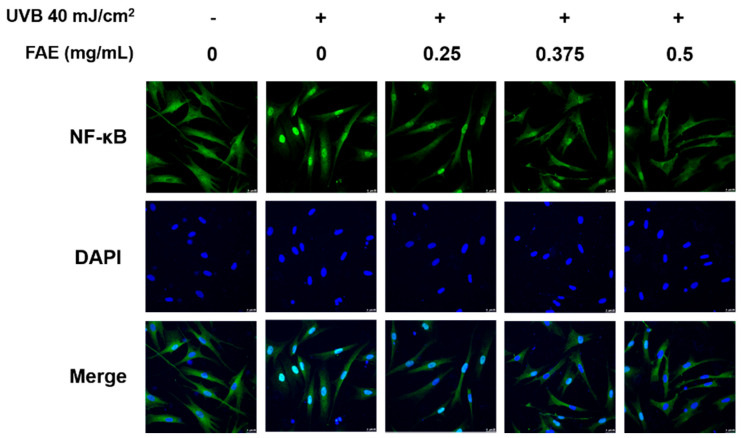
The effects of *Ficus awkeotsang* Makino extract (FAE) on UVB-irradiated nuclear translocation of NF-κB in Hs68 cells.

**Figure 5:**
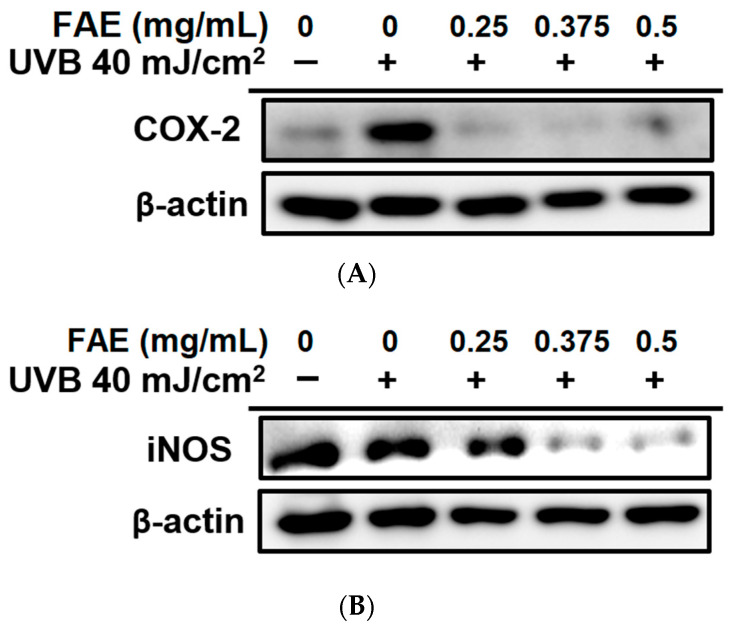
The effects of *Ficus awkeotsang* Makino extract (FAE) on UVB-irradiated protein expression of (**A**) cyclooxygenase-2 (COX-2), and (**B**) inducible nitric oxide synthase (iNOS) in Hs68 cells.

An immunofluorescence analysis was also performed to determine whether the expression of NFκB in the nucleus decreased upon FAE treatment. The expressions of NFκB and DAPI in HS68 cells after treatment with different FAE dosages (0.25 to 0.5 mg/mL) after UVB irradiation were determined. Expression levels of DAPI were approximately the same in all treatments, while a clear decrease in NFκB expression level was observed following the addition of 0.25–0.5 mg/mL of FAE in the culture medium, when compared to the UVB-irradiated treatment only (Figure 4). We also aimed to identify the mechanism by which FAE suppresses the expression inflammatory mediators. The effects of FAE on the expression of cyclooxygenase-2 (COX-2) and inducible nitric oxide synthase (iNOS) were also determined (Figure 5A,B). The expression of these inflammatory signaling molecules were stimulated with 40 mJ/cm^2^ UVB. However, the expression levels of COX-2 and iNOS were found to decrease significantly when FAE concentration was increased from 0.25 to 0.5 mg/mL, and from 0.325 to 0.5 mg/mL, respectively. 

### 3.4. In Vivo Inflammatory Action

**Figure 6:**
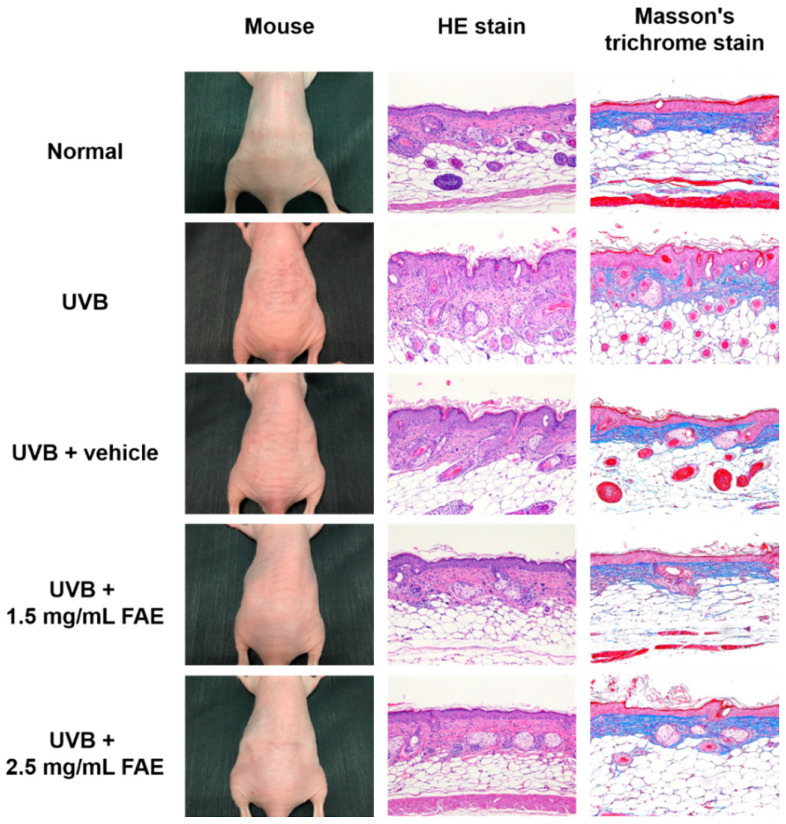
Analysis of the inflammatory response in BALB/c nude mice upon *Ficus awkeotsang* Makino extract (FAE) treatment following UVB irradiation. Images of dorsal skin, HE staining, and Masson’s trichrome staining of the skin area after 10th week.

We investigated the effect of FAE on skin inflammation following chronic exposure to UVB irradiation for 10 weeks using a BALB/c nude-mouse model. Redness was induced by UVB irradiation in BALB/c nude mice when applied alone or in combination with PEG (vehicle) treatment (Figure 6). To further assess the degree of inflammation, we examined sections of the dorsal skin tissue using H&E staining, and evaluated the extracellular matrix composition in the tissue using Masson’s trichrome staining. Figure 6 shows that epidermal thickness slightly increased upon UVB irradiation, yet decreased upon the application of 1.5 to 2.5 mg/mL FAE in nude mice. Masson’s trichrome staining revealed the presence of widespread and densely-packed collagen on the dermal skin with 1.5 mg/mL and 2.5 mg/mL FAE treatments. We also observed a decrease in collagen level and accumulation of inflammatory cells in the dorsal skin tissue following UVB irradiation and vehicle treatment.

**Figure 7:**
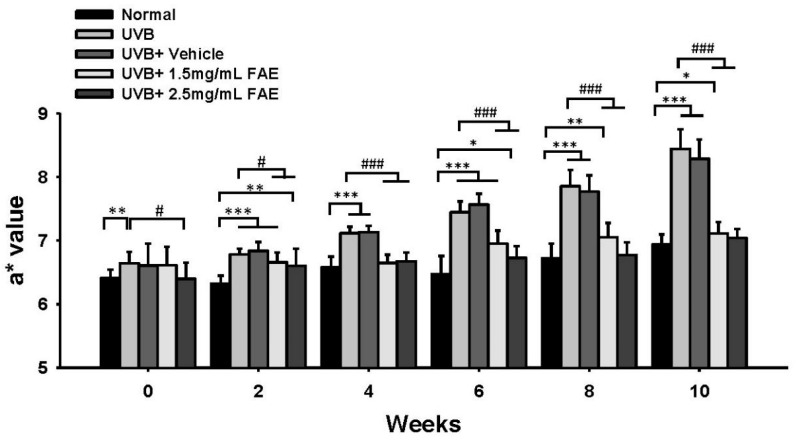
Quantification a* value of dorsal skin upon *Ficus awkeotsang* Makino extract (FAE) treatment at different concentrations following UVB irradiation on BALB/c nude mice from 0 to 10 weeks. Statistically significant differences from the control were observed with * *p* < 0.05, ** *p* < 0.01, *** *p* < 0.001; statistically significant difference from UVB-exposure group was observed with # *p* < 0.05, ### *p* < 0.001 statistical significance was determined using Tukey’s HSD test. *n* = 3; all error bars represent the standard deviation of the mean.

We further assessed the redness response of the dorsal skin of nude mice by determining the a* value, which serves as an inflammatory biomarker (Figure 7). Continuous UVB irradiation and vehicle treatment increased the a* value of epidermal skin from 7.3 to 21.7% during a two-to-ten-week period compared to the non-irradiated control. In contrast, supplementation with 1.5 mg/mL FAE after UVB irradiation yielded significant (*p* < 0.001) differences in the a* value ranging from 7.1% to 19.2% with that of the non-irradiation control during a four-to-ten-week period. Statistically significant (*p* < 0.001) levels of suppression (more than 6.7%, 11.1%, 16.1%, and 20.2%) of the redness of murine skin were detected after 2.5 mg/mL FAE treatment after UVB irradiation during a four-to-ten-week period. However, no significant difference was observed in the a* value between 1.5 mg/mL and 2.5 mg/mL FAE treatment. These results indicate that FAE is an effective anti-inflammatory agent following skin injury induced by UVB irradiation. A relatively low FAE dose (1.5 mg/mL) was sufficient to inhibit the inflammatory response following UVB irradiation.

**Figure 8:**
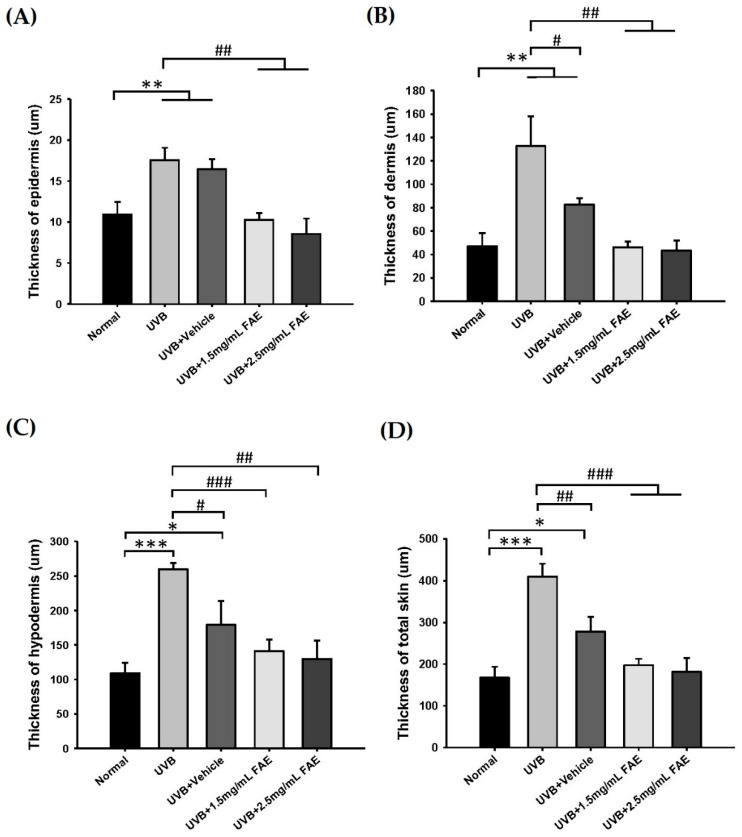
Quantification thickness of dorsal skin of BALB/c nude mice upon *Ficus awkeotsang* Makino extract (FAE) treatment at different concentrations following UVB irradiation for 10 weeks. (**A**) Thickness of epidermis, (**B**) thickness of dermis, (**C**) thickness of hypodermis, and (**D**) thickness of total skin. Statistically significant differences from the control were observed with: * *p* < 0.05, ** *p* < 0.01, *** *p* < 0.001; statistically significant difference from the UVB-exposure group was observed with: # *p* < 0.05, ## *p* < 0.01, ### *p* < 0.001 statistical significance was determined using the Tukey’s HSD test. *n* = 3; all error bars represent the standard deviation of the mean.

**Figure 9:**
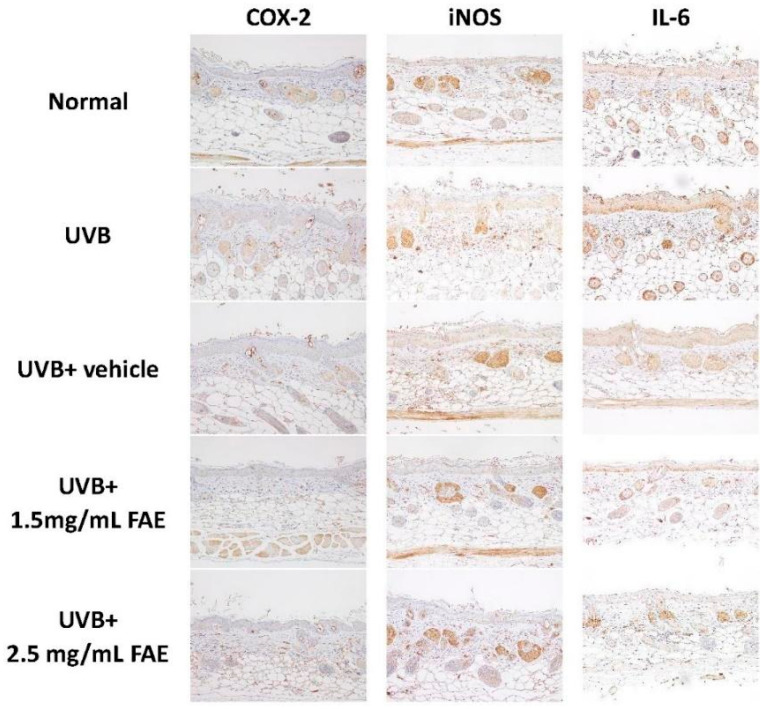
The inhibitory effects of *Ficus awkeotsang* Makino extract (FAE) in BALB/c nude-mice dorsal skin after UVB irradiation. The protein expression after different treatments were determined immuno-histologically by staining the tissue sections blotted with the COX-2, iNOS, and IL-6 antibody.

The thickness of skin layers of nude mice was measured (Figure 8). After UVB irradiation, the thicknesses of the epidermis, dermis, hypodermis, and total skin increased significantly (*p* < 0.001) by 60.9%, 181.5%, 137.6%, and 127.6%, respectively, compared to that of the non-irradiated control. However, thicknesses of skin layers showed no difference with respect to the non-irradiated control upon FAE treatment at 1.5 mg/mL and 2.5 mg/mL. Immunohistochemical localization analysis (Figure 9) clearly revealed the expression of COX-2, iNOS, and Interleukin-6 (IL-6) in the epidermis and dermis skin tissue of nude mice treated with UVB and vehicle. Interestingly, a similar level of inhibition of inflammation-related protein expression was displayed at low (1.5 mg/mL) and high (2.5 mg/mL) FAE doses, which indicates a dose-dependent effect of FAE for tissue healing upon UVB damage.

### 3.5. Identification of Antioxidant Compounds in FAE

Antioxidant characteristics of plant extracts arise as a result of complex processes involving several mechanisms. We focused on two classes of plant-derived secondary metabolites, phenols, and flavonoids, as indicators of antioxidant presence in FAE, as they play important roles in redox processes in plants. We determined the total phenol content of the seed extracts using a modified Folin–Ciocalteu reagent method. The total phenol content was determined to be 107.6 mg GAE g^−1^ DW using a calibration curve constructed using gallic acid as the standard (regression coefficient = 0.9978). We also determined the total flavonoid content using the modified AlCl_3_ method. The total flavonoids content was determined to be about 10.3 mg QE g^−1^ DW using a calibration curve (regression coefficient = 0.9963) constructed using quercetin. The amounts obtained using this curve were thus expressed in terms of quercetin equivalents (QEs) per gram of dry extract weight. The flavonoids/phenol ratio of FAE was found to be 0.1 (Table 1).

**Figure 10:**
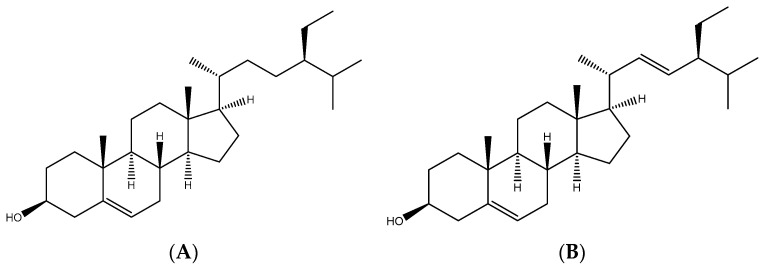
Analysis of chemical constituents of the *Ficus awkeotsang* Makino extract. (**A**) Structure of β-sitosterol and (**B**) structure of stigmasterol.

Finally, nuclear magnetic resonance spectroscopy (^1^H-NMR) and high-performance liquid chromatography (HPLC) were used to determine the primary chemical structure of FAE. As seen in Supplementary Figure S1, the data clearly exhibit two distinct sets of major peaks (ppm) at 4.79 ppm and 3.81 ppm, which might include the solvent residual signal (i.e., HOD in D2O) and other mixture compounds. In addition, the rest of the peaks are overlapped, and difficult to garner any specific information. Based on data from previous studies [33,34], these peaks were further analyzed by a comparison with authentic samples through HPLC (data no showed). The HPLC spectra of FAE peaks were only similar to those of β-sitosterol (Figure 10A) and stigmasterol (Figure 10B). The presence of a single/double bond between C22 and C23 was the only difference between the two compounds: a C22=C23 double bond was present in stigmasterol, whereas a single bond was present in β-sitosterol. However, the identification of sterol components in FAE should be further separated by mass spectrometry in the next study.

## 4. Discussion

Reactive oxygen species (ROS) include radical and non-radical oxygen species, such as superoxide anions (O_2_^−^), hydrogen peroxide (H_2_O_2_), and hydroxyl radicals (HO^•^), which are formed during mitochondrial oxidative metabolism as well as cellular immune responses. ROS have evolved as regulators of intracellular signaling pathways [35,36]. ROS production in the human body increases abnormally in the presence of psychiatric disorders or physiological disease [37]. Excessive ROS production in response to oxidative stress causes molecular damage and inflammatory responses and has been linked to various chronic diseases, including diabetes, cardiovascular diseases, eye disorders, arthritis, obesity, neurodegeneration, and cancer [38,39]. Therefore, the use of various antioxidants for medical purposes have been widely discussed over the last decade, owing to their ability to scavenge excess ROS and anti-inflammatory effects [40,41]. Several synthetic antioxidant compounds have been developed as food additives or dietary supplements in the past. However, the clinical usefulness of the chemicals is limited due to toxicity, high cost and side effects. For example, butylated hydroxyanisole (BHA) and butylated hydroxytoluene have been shown to cause liver, stomach, thyroid, and kidney problems and even carcinogenesis [42,43]. On the other hand, exogenous natural antioxidants that are mainly derived from edible and medicinal plants are considered relatively safe with no side effects, and their production is more affordable than synthetic chemicals [44]. Issues, such as lack of availability, difficulties of large-scale production, and potential cumulative effects still need to be evaluated before natural free radical scavenger molecules, especially those obtained from non-traditional food or stranger plants, can be considered for use in commercial applications [45].

### 4.1. Antioxidant Compounds in FAE

Antioxidant effects arise as a result of a complex process that involves several intracellular pathways. Thus, multiple types of antioxidant capacity analyses should be performed to evaluate various types of antioxidant action [46]. Five types of tests that complement each other were used in this study to assess the antioxidant activity of FAE. According to the results, FAE exhibited a strong ability to scavenge DPPH, H_2_O_2_, and hydroxyl radicals, similar to the scavenging abilities of neutral ascorbic acid, mannitol, and synthetic BHA. FAE was also found to have a high antioxidant capacity via ferrous chelating activity as well as reducing capability. Such high DPPH radical scavenging, reducing capacity, and ferrous ion chelating activity of the residue from jelly fig was also observed in a previous study [47]. Intracellular ROS levels were also significantly reduced upon FAE treatment following UVB irradiation in human cells. These findings indicate the presence of various mechanisms [48]. For instance, the H_2_O_2_ assay is based on the content of the oxidizing agent, whereas DPPH assays are based on electron and H-atom transfer, and the ferrous chelating assay is based on the Fe^2+^ transfer in a single-electron reaction [49,50,51].

To date, there are no previously separated components for jelly fig extract. Therefore, we first performed a pilot test (data no showed) and the result showed that the bio-activities of individual separated compounds were weaker than those of total jelly fig extract (FAE). The biological activity may be contributed to by the mixture of FAE. However, several studies have shown that flavonoids and phenolic compounds present in plants mainly contribute to their antioxidant activities [52,53,54]. Phenolic compounds are also important secondary metabolites that protect against herbivores and pathogens, and thereby facilitate cell survival [55,56]. Here, two antioxidants were identified from jelly fig. The flavonoid content of FAE was also similar to those of fig extracts [57], whereas the total phenol content was found to be the highest among those of other *Ficus* species. The total phenol content of FAE was 5- to 200-fold higher than those of fig extracts of the leaves, seeds, or fruit. Furthermore, the DPPH scavenging activity of FAE was approximately 2- to 480-fold higher than that of another *Ficus* species previously studied [58,59,60,61]. Free radical scavenging activity is often negatively correlated with cytotoxicity of compounds, e.g., fig extract (IC_50_: 10–19 μg/mL), gallic acid (IC_50_: 20.86 μg/mL), and BHA (IC_50_: 6.42 μg/mL) [62,63,64]. However, FAE was not found to show any cytotoxicity in Hs68 cells. In contrast, FAE showed good in vitro biocompatibility and promoted cell viability.

### 4.2. Anti-Inflammatory Compounds in FAE

Inflammation is an innate immune response that can be triggered by a variety of homeostatic changes, such as autoimmune reactions, infection, injury, toxicity, and disease [65,66]. The UV-induced inflammatory activity in skin is often triggered upon sun exposure, and leads to damage in the skin, such as changes in skin thickness, aging, redness, wrinkles, and even tumorigenesis [67,68]. An imbalanced antioxidant and free radical content may also induce oxidative stress and further trigger proinflammatory signaling cascades in the cell and development of inflammation-associated diseases [69,70,71]. To evaluate the anti-inflammatory effect of FAE on UVB-irradiated skin, we first determined the effects of different FAE doses on HS68 cells. Although, the levels of NFκB, COX-2, and iNOS were found to decrease upon FAE treatment, the dose of FAE (between 0.25 and 0.5 mg/mL) had no clear effect in vitro. However, the dose of FAE was nevertheless found to be highly correlated with longer-term anti-inflammatory effects in BALB/c nude mice. Other properties, including redness, skin thickness, and inflammatory cell accumulation, were similarly decreased in the experimental group with different FAE doses. The a* value of mouse skin was significantly reduced (i.e., by more than 20%, *p* < 0.001) in the presence of FAE, compared to that obtained with the vehicle treatment or control after UVB irradiation. This finding is in good agreement with those of several previous studies, in which the antioxidant of plant extracts were also linked to the inhibition of inflammatory-related activity in skin [70,72,73]. Further analysis showed that the levels of inflammatory proteins COX-2, iNOS, and IL-6 decreased in the skin, especially in the epidermis and dermis. These results strongly suggest that FAE is not only an effective antioxidant, but also an outstanding anti-inflammatory agent after chronic UVB exposure.

We further identified the HPLC spectrum of FAE was similar to that of β-sitosterol or stigmasterol. Although the compounds of those peaks remain unclear and require further confirmation by LCMS, it is worth mentioning that similar anti-inflammatory action was displayed in phytosterols and FAE. The β-sitosterol or stigmasterol are bioactive phytosterols found in many plant species [74]. β-sitosterol was previously linked to chronic obesity-related inflammation, rheumatoid inflammation, and ovalbumin-induced airway or lung inflammation in mice [75,76,77,78,79]. Stigmasterol was also implicated as an inhibitor in ovalbumin-induced airway inflammation, lipopolysaccharide-induced lung inflammation, and rheumatoid arthritis [80,81,82]. Our findings confirm the anti-inflammatory effects of FAE on UVB-induced inflammation. Our results also demonstrate that inflammation is alleviated by inhibiting NF-κB and several pro-inflammatory mediators [83]. Thus, we suggest that, in addition to polyphenols, other compounds (ie, phytosterol compounds, β-sitosterol, and stigmasterol) may also contribute to the strong anti-inflammatory properties of FAE.

### 4.3. Molecular Mechanism of FAE

**Figure 11:**
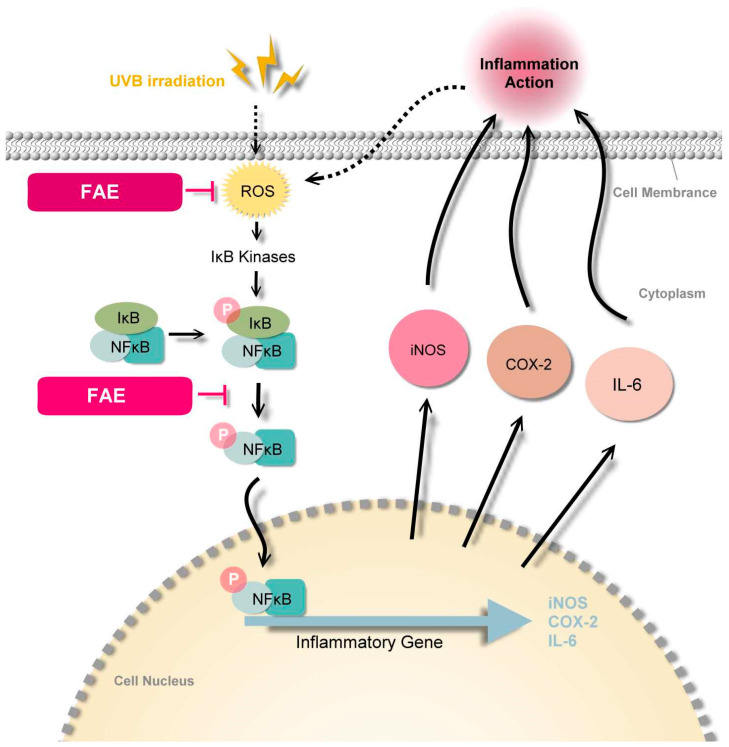
Schematic representation of inhibition of the inflammation-related signal transduction pathway by *Ficus awkeotsang* Makino extract (FAE). ROS, reactive oxygen species; IκB, inhibitor of κB; and NFκB, nuclear factor kappa B. The ROS are produced after UVB irradiation, while the IκB kinase activates the NFκB pathway. Activation of the NFκB triggers expression of several inflammatory genes, including members of the iNOS (inducible nitric oxide synthase), COX-2 (cyclooxygenase-2), and IL-6 (interleukin-6). iNOS and COX-2 regulate the inflammatory response. IL-6 is a multifunctional cytokine that regulates the immune response.

Nuclear factor-κB (NF-κB) is a key transcription factor in inflammatory cascades due to its ability to induce transcription of proinflammatory genes [84]. The cytoplasmic form of NF-κB is inactive and is associated with inhibitors of κB (IκB) [85]. Photodamage to the skin, leukocytes, and mast cells are observed in damaged skin tissue, and lead to an increased uptake of ROS. When the production of ROS overwhelms the cellular antioxidant capacity, it contributes to oxidative stress, then mediates the upstream kinase of the NFκB pathway. Thus, an increased phosphorylation of the NF-κB–IκB complex was observed [86,87,88,89]. Immunofluorescence analysis of Hs68 cells confirmed that the expression of NF-κB increased after UVB irradiation, and further revealed that FAE downregulated NF-κB expression levels, whereas similar DAPI expression levels were observed across the experiments with different FAE treatments doses. Several studies have previously demonstrated that the ubiquitinated NF-κB dimer is released from the cytoplasm and translocated to the nucleus to promote inflammation-related protein transcription and further induce inflammation [90,91]. Our study provides strong evidence that levels of COX-2, iNOS, and IL-6 decrease in the presence of ROS and NF-κB, which are inhibited by the *Ficus awkeotsang* extract. This was followed by the downregulation of the proinflammatory signal transduction pathway, eventually leading to an anti-inflammatory response in nude mice (Figure 11).

## 5. Conclusions

To our knowledge, this is the first study to identify the major bioactive components of FAE responsible for antioxidative and anti-inflammatory activities. FAE also exhibited significant antioxidative and anti-inflammatory properties without any cytotoxicity. Our data demonstrated that FAE function as suppressors of UVB-induced inflammatory action by downregulating iROS and NF-κB. In summary, our results suggest that FAE is a non-toxic substitute for synthetic antioxidants used in food, cosmetics, and pharmaceutical applications.

## Figures and Tables

**Table 1:** Total phenol and flavonoids contents, flavonoids/phenol ratio, and extraction yields from the *Ficus awkeotsang* Makino extract.

Total Phenol and Flavonoids Contents	Jelly Fig Extract
Total phenol content (mg GAE g^−1^ DW)	107.622 ± 0.176 ^z^
Total flavonoids content (mg QE g^−1^ DW)	10.251 ± 0.020
Flavonoids/phenol ratio	0.10

^z^ Mean ± standard deviation of the mean (n = 3).

## Data Availability

Data is contained within the article.

## Supplementary Materials

The following are available online at: https://www.mdpi.com/article/10.3390/antiox11050981/s1, Figure S1. Analysis of chemical constituents of the *Ficus awkeotsang* Makino extract by Nuclear magnetic resonance spectroscopy (NMR) spectrum.
