# Supplementary material for: Jelly Fig (Ficus awkeotsang Makino) Exhibits Antioxidative and Anti-Inflammatory Activities by Regulating Reactive Oxygen Species Production via NFκB Signaling Pathway
Source: Antioxidants (Basel). 2022 May 17;11(5):981. doi: 10.3390/antiox11050981 (PMC9138086; doi:10.3390/antiox11050981)
Supplement: Supplementary file 1 [file antioxidants-11-00981-s001.zip › antioxidants-1711102-supplementary.pdf]

antioxidants

MDPI

Supplementary Materials: Jelly Fig (*Ficus awkeotsang* Makino) Extract Exhibits Antioxidative and Anti-Inflammatory Activities by Regulating Reactive Oxygen Species Production via NFκB Signaling Pathway

Meng-Jin Lin , Ping Lin , Kuo-Ching Wen , Hsiu-Mei Chiang and Mei-Chun Lu

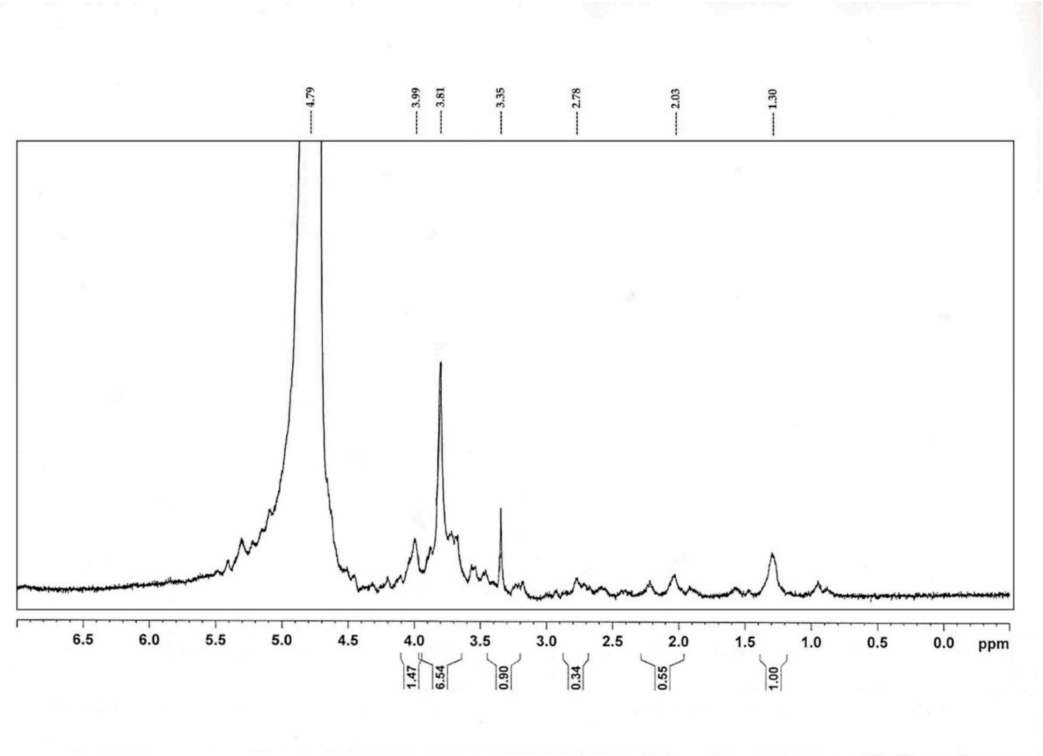

Figure S1. Analysis of chemical constituents of the Ficus awkeotsang Makino extract by Nuclear magnetic resonance spectroscopy (NMR) spectrum.
